# Numerical and Experimental Mechanical Analysis of Additively Manufactured Ankle–Foot Orthoses

**DOI:** 10.3390/ma15176130

**Published:** 2022-09-03

**Authors:** Ratnesh Raj, Amit Rai Dixit, Krzysztof Łukaszewski, Radosław Wichniarek, Justyna Rybarczyk, Wiesław Kuczko, Filip Górski

**Affiliations:** 1 Department of Mechanical Engineering, Indian Institute of Technology (Indian School of Mines), Dhanbad 826004, Jharkhand, India; 2 Faculty of Mechanical Engineering, Poznan University of Technology, Piotrowo 3 STR, 61-138 Poznan, Poland

**Keywords:** 3D printing, carbon fiber, polylactic acid (PLA), ankle–foot orthoses (AFO), mechanical analysis, simulation

## Abstract

Growing age and different conditions often require the replacement of orthoses, and FDM-based 3D printing can produce them quickly with less investment. In today’s market for orthotics, these characteristics are highly desired. Therefore, this study is fully focused on the optimization and strength analysis of FDM 3D-printed ankle–foot orthoses (AFO) fabricated using PLA and PLA reinforced with carbon fiber (PLA-C). An increase in ankle plantar-flexor force can be achieved by reinforcing thermoplastic AFOs with CFs. Specially designed mechanical strength tests were conducted at the UTM to generate force–displacement curves for stored elastic energy and fracture studies. The mechanical behavior of both AFOs was predicted with the help of an FEA. The model predictions were validated by comparing them with mechanical strength testing conducted under the same loading and boundary conditions as the FEA. In both the prediction and experimental analysis, the PLA-C-based AFOs were stiffer and could withstand greater loads than the PLA-based AFOs. An area of high stress in the simulation and a fracture point in experimentation were both found at the same location. Furthermore, these highly accurate models will allow the fabrication of AFOs to be improved without investing time and resources on trials.

## 1. Introduction

An ankle–foot orthosis (AFO) is a medical device that is worn externally and supports the foot, ankle, and lower leg to treat lower limb problems such as unsteadiness, foot drop, and bony foot abnormalities [1]. They are frequently recommended for children and adults with neurological illnesses such as cerebral palsy [2], cerebrovascular accidents [3], Charcot–Marie–Tooth disease [4], and multiple sclerosis [5] to improve their walking abilities. The use of customized AFOs is frequently prescribed to minimize trips and falls due to foot drop, reduce the chronic pain and fatigue caused by joint deformities, and reduce the fatigue caused by controlling the ground reaction force experienced during the stance phase of the gait [6]. There are many complaints from AFO users about poor fit, pain, discomfort, appearance, design, and the limitations in accompanying footwear choices. According to research, children and teenagers, females, and persons who live alone are the most dissatisfied AFO users [7]. Consequently, most of the children and adults with musculoskeletal and neuromuscular abnormalities do not use their recommended AFOs during gait and instead use compensatory techniques, which are physiologically inefficient and potentially hazardous [8]. Thus, many patients only wear their devices once they develop severe symptoms, even though earlier use of an AFO may have significant clinical benefits.

AFOs are manufactured from plaster of Paris casts of the patient’s lower legs in the majority of cases [9]. The actions that must be taken in order to create a unique an AFO are as follows: the patient’s ankle and foot are measured, a positive plaster model is made using casting technology based on the negative plaster imprint, the positive plaster model is modified to fit the patient’s anatomy, and the AFO is vacuum-thermoformed and fitted. The labor-intensive process, limited design options, expense, and long waiting times are some of the downsides of this traditional approach [10,11]. These downsides are leading the world to discover new techniques for making customized AFOs [12]. Various additive manufacturing (AM) technologies have been adopted for the replacement of the conventional methodology [13,14,15].

The process of AM involves joining materials in a rapid layering process in order to form functional three-dimensional (3D) objects [16,17]. There are seven different basic AM mechanisms currently in use for the production of these functional objects. Material extrusion, binder jetting, material jetting, direct energy deposition, powder bed fusion, vat photopolymerization, and sheet lamination are the widely accepted AM mechanisms [18]. All these mechanisms have various technologies to perform 3D printing, e.g., digital light processing, selective laser sintering, and fused deposition modeling (FDM), and can eliminate several steps from the conventional processes for making AFOs [19].

The 3D printing mechanisms allow for design freedom by permitting alteration from traditional design frameworks and thus facilitate the development of patient-specific AFOs. Furthermore, since 3D printing does not require specialized tooling and unit costs are independent of batch size, it favors the production of individualized items in one piece. The AFOs can be tailored to meet specific biomechanical needs in order to enhance functions, fitting, and aesthetics. Patient-specific fabricated AFOs using 3D-printing are expected to significantly improve patient satisfaction, AFO usage compliance, and other health-related results [7,11,19,20].

Among all available 3D printing technologies, FDM is the most popular because of its easy handling, availability, and cost-effectiveness. A thermoplastic polyurethane-based filament has been used to manufacture AFOs and has shown remarkable results compared with traditional ones [21]. Taking it to the next level, 3D-printed AFOs are equipped with additional spring joints [22]. Hence, 3D printing allows for more customizing options, and it should be explored further to obtain better AFOs. A number of design modifications have also been made while using FDM as an AFO manufacturing process. In such applications, thermoplastics have shown some limitations in terms of strength, which is why researchers began with mixed thermoplastics and reinforcements in thermoplastics. It was found that polylactic acid (PLA) is not solely able to meet the required strength for AFOs. Researchers then used PLA with nylon-12 as the printing material to meet the required strength [23]. Producing AFOs with two materials in different layers has also been investigated to achieve a high strength. Dual-nozzle FDM 3D printers have been employed for this since they facilitate the process of using two materials together [24]. Conventionally prepared AFOs are mostly modified by adding carbon fiber (CF), and they exhibit a remarkable increase in strength [25]. Therefore, CF-reinforced thermoplastics usage would allow a considerable strength gain in the manufacturing of AFOs to be obtained through the FDM technique. CF has been used as a reinforcement material for FDM 3D-printed samples in various applications to increase strength [26,27]. A 7% volume fraction of CF in ABS printed using FDM has demonstrated a fine increment in tensile strength [26]. PETGs containing different concentrations of CF processed by FDM possess considerable tensile, compression, and flexural strengths [28]. In most research studies about 3D-printed AFOs, researchers performed their own method of testing their mechanical strength. In most of them, the test influenced by bending tests of samples was adopted [14,29]. To obtain the deflection and fracture testing, they simply fixed one section of the AFO and applied a force at the other end. The compression tests of the foot section have also been examined by various researchers. In both cases, the deflection test influenced by the bending test more realistically resembles the original scenario of AFO usage.

Finite element analysis (FEA) modeling has recently been applied by researchers to simulate and optimize the operating performances of AFOs [30,31]. An entirely discrete series of parameters is used to properly describe and regulate the design of a computer-aided design (CAD) model. An FEA of CAD models allows for the prediction of in situ stresses and strains by using boundary conditions that replicate real-world conditions [32,33]. FEA has been used to analyze and develop a range of devices in the prosthetics and orthotics field. Additionally, FEA can estimate stress distribution, material deformation trends, and the orthosis dimensions required to replicate the design features of commercial orthoses. It may be possible to quickly determine the ideal AFO functional features, such as bending stiffness, by using FEA of fully parameterized CAD models. A number of times, the bending strength of FDM 3D-printed AFOs has been examined using the FEA software ANSYS workbench [13,30]. To predict the actual mechanical behavior of the AFOs, researchers have considered the von Mises stress distribution and deformation data. AFOs based on PLA have been evaluated using ANSYS simulation and are being replaced with nylon-12 during production in high-stress-concentration areas to attain a higher strength [23]. In order to predict the optimal neck size of the lower section of AFOs, different neck sizes of the lower section were considered in the simulation [14].

PLA is the most common cheap and biocompatible thermoplastic [34]. Thus, in this study, PLA- and PLA reinforced with CF (PLA-C)-based FDM-printed AFOs are examined in terms of mechanical strength. The authors have selected PLA as an initial material, as they strive for a low-cost, widely available manufacturing process, with the possibility of recycling of used materials. Elastic energy has been used for the analysis of the results. An Abaqus-based FEA simulation is also shown to facilitate a better analysis of sample deflection and fracture.

## 2. Materials and Methods

### 2.1. Research Concept and Plan

No previous known literature addresses use of 3D-printed filament composite materials in the production of leg orthoses for children or the mechanical properties testing of such products (not just samples). Moreover, no previous known literature makes a comparison between the experimental and simulated (FEA) results of mechanical strength of such orthoses, including the use of composite (CF-reinforced) materials. As such, to gather new knowledge, a certain set of experiments and analytical studies were realized.

The objective of the current research is to examine which AFO, PLA or PLA-C, performs well in terms of mechanical strength. An analysis of the behavior deviation of CF-reinforced PLA AFOs compared with PLA-based ones has been conducted. Therefore, the ultimate goal of this research is to explore how different materials impact AFO strength after additive manufacturing. In order to save time and resources on experimental trials, FEA simulations were included in the plan to predict the behavior of both types of AFOs.

In common practice, materials are classified in terms of force-displacement (*F*-*d*) curves. These curves are derived from experimental testing. With this relationship between force and displacement, elastic energy (*E*) can be calculated using Equation (1) [25].

(1) $$E=\left(\frac{1}{2}\right)F\times d$$

This elastic energy calculation approach has been adopted to analyze the AFOs’ deformation behavior under loading conditions. The von Mises stress distribution and the deformation distribution plot of the simulation were used to compare the experimental results.

Most 3D-printed complex shapes have different modulus values than standard samples because the internal geometry is generated by the layering of materials, which makes the structure different than the standard samples. Therefore, Equation (2) was used for real modulus calculations and integrated into the dual simulation process (described later) in order to make the most precise prediction. The equation was derived from Hooke’s law of proportionality and was also used in previous studies related to orthoses [35]:

(2) $$E_{e}=\left(d_{s}\times E_{s}\right)/d_{e}$$

where *E_e_* is the real elastic modulus value (MPa); *E_s_* is the elastic modulus (MPa) of the standard sample; *d_e_* is the displacement value obtained experimentally (mm); and *d_s_* is the displacement value obtained in the first simulation (mm).

PLA (Spectrum premium filaments, Spectrum Group, Sosnowiec, Poland) and PLA-C (F3D filament, FINNOTECH, Katowice, Poland) filaments were used in the study. According to the manufacturer, the PLA-C filament contained 10% carbon fibers.

### 2.2. Design and Customization of Orthosis Model

**Figure 1:**
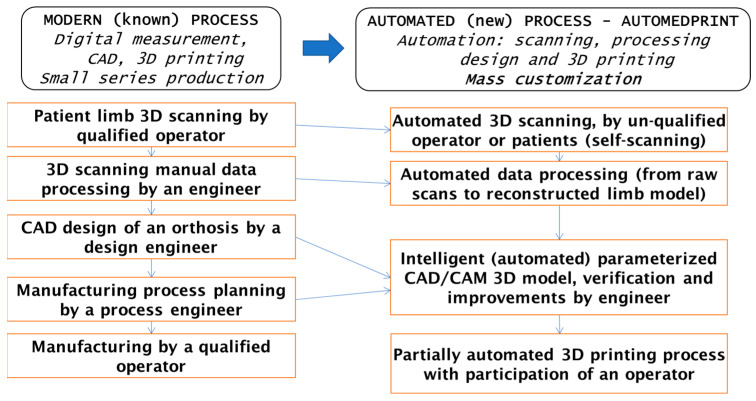
AutoMedPrint system: assumptions for process automation in comparison with a typical process.

In order to create the AFO model for the 7-year-old female patient, the AutoMedPrint system (developed at Poznan University of Technology, prototype version created by some of the authors in previous work, available only for internal use) was used. The system allows the realization of a modern, digitally oriented process of design and manufacturing of orthopedic and prosthetic devices, as shown in authors’ earlier works [36]. The operation of the system is partially or completely automated (depending on the stage), and the main assumptions of automation are presented in Figure 1. This is a novelty in comparison to the existing literature, where automation is usually only considered or shown as the main point of the studies. The authors made use of a new methodology, implemented in form of a unique prototype system.

**Figure 2:**
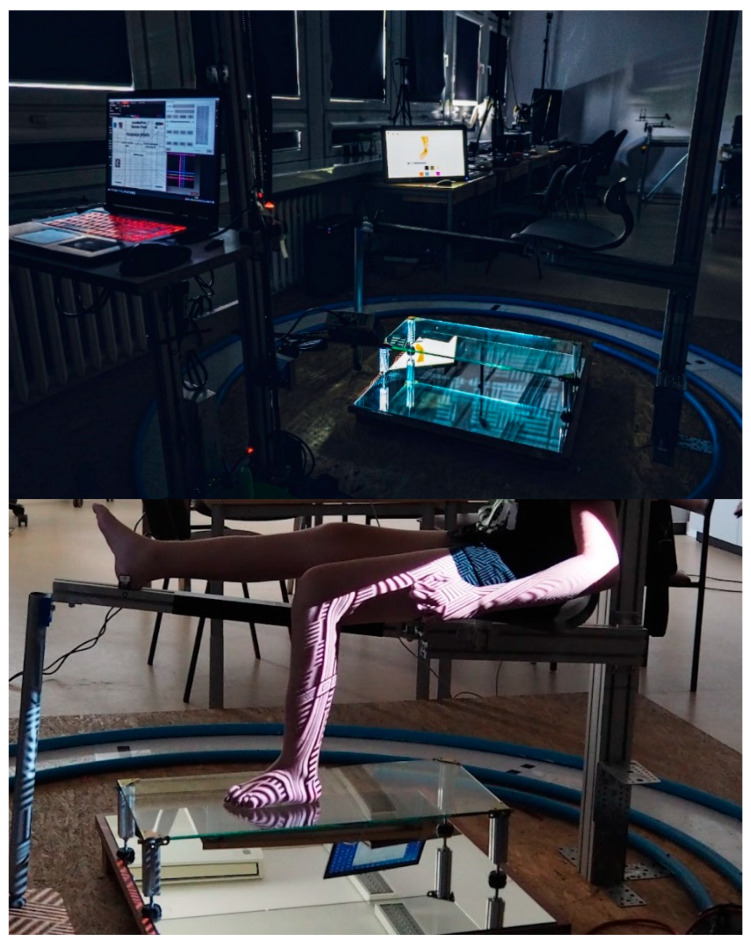
AutoMedPrint system: stand for lower limb scanning and the leg scanning process (automedprint.put.poznan.pl, access on 15 July 2022).

**Figure 3:**
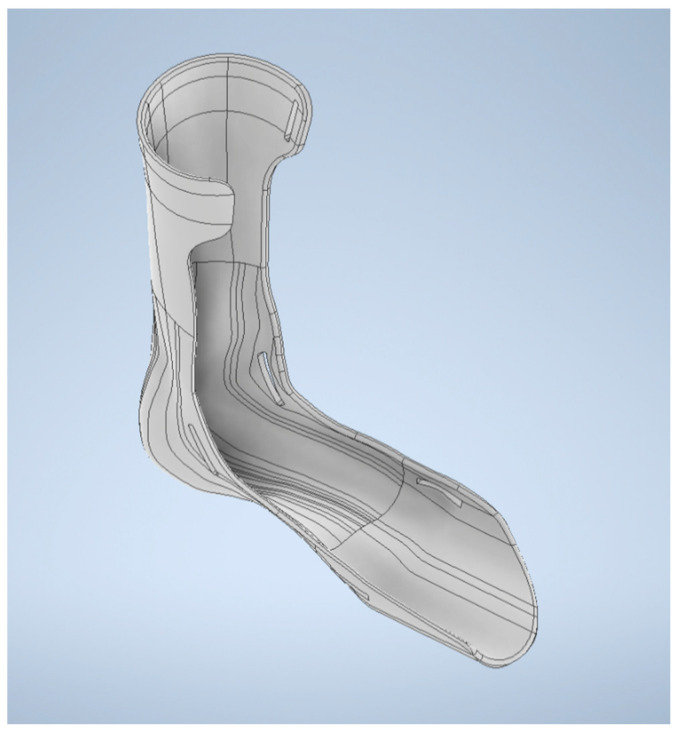
Generated AFO after scanning, design, and modification.

First, an optical scanner, the David SLS-3 (David vision systems GmbH, Koblenz, Germany), was used to scan the patient’s right lower limb on a specially designed workstation (Figure 2). A total of six scans were conducted in multiple directions in order to obtain a better model. An automated algorithm for the processing of 3D scan data was developed by using MeshLab software. The alignment of scans, the removal of redundant information, and the transformation of scans into a correct global coordinate are some of the steps that occurred during this process. A set of points was obtained by constructing sections of the plane series. From these points, splines were generated, which, when combined with multisection extrusion and offsets, created the main shape of the orthosis. The automated algorithm in VBA (Visual Basics for Application) was used initially to preselect the points in an excel spreadsheet. The data were fed to a 3D model in AutoDesk Inventor software (version 2022) after being processed by the automated algorithm. The process has been described in previous publications [36]. The AFO model that was generated after scanning and modifications is illustrated in Figure 3. The model was verified for functional and therapeutical correctness by biomedical engineers and a physiotherapist.

### 2.3. Fixture Formation and Design Modification for Strength Testing

Strength checks were conducted using fixtures on a traditional universal testing machine (UTM). The fixture was made from mild steel in a manner that the lower half of the AFO could be screwed into it. A load-handling fixture was designed and 3D-printed for the uniform distribution of the load throughout the circumference of another end AFO since the force generation point of the bending test setup is flat (with a cylindrical roller). One additional filler support was designed and printed in PLA to fill the gap between the mild steel fixture and the AFO. Due to the fixation being at the lower foot part of the AFO, a small part of the foot was cut apart, and three holes were made in the remaining material for attaching it to a mild steel AFO holder and gap fillers.

### 2.4. The 3D Printing of AFOs

The modified AFO with its holes and cut section was manufactured using an FDM-technology-based 3D printer (M200 plus, Zortrax S.A., Olsztyn, Poland). For each material, PLA and PLA-C, three samples of AFOs were manufactured. For printing these AFOs, extrusion temperatures of 210 °C were chosen for PLA and 215 °C for PLA-C, along with 100% infill. The orientation of the layers (Z axis) was consistent with the natural orientation of the orthosis during its usage. Various printing parameters and weights of the printed samples are listed in Table 1.

### 2.5. Mechanical Testing

**Figure 4:**
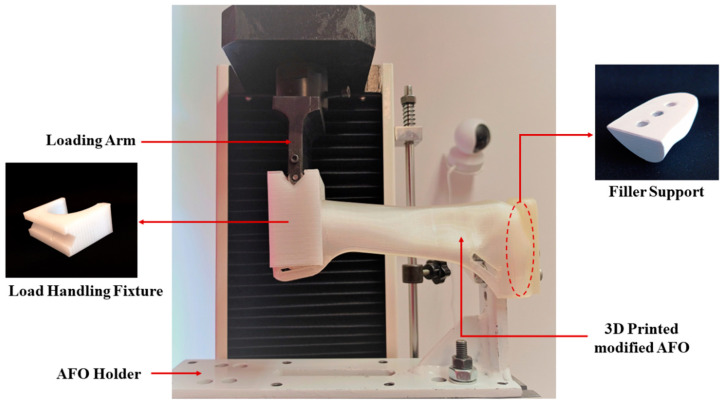
Strength testing setup at the UTM.

The 3D-printed AFOs of PLA and PLA-C were mounted one by one on the mild steel AFO holder with gap filler support and screws. Measurements were taken using a UTM (SUNPOC, Guiyang, China) at room temperature and a crosshead speed of 8.0 mm/s. The load-handling fixture was attached to the upper end of the AFO so that loads could be applied there. A load of ~0.8 times the weight of the patient was chosen for the testing, which is 160 N. To ensure accuracy and reproducibility, three AFO samples of each type were tested. A picture illustrating the assembly of the test setup is shown in Figure 4.

### 2.6. Finite Element Analysis (FEA) Modeling

**Figure 5:**
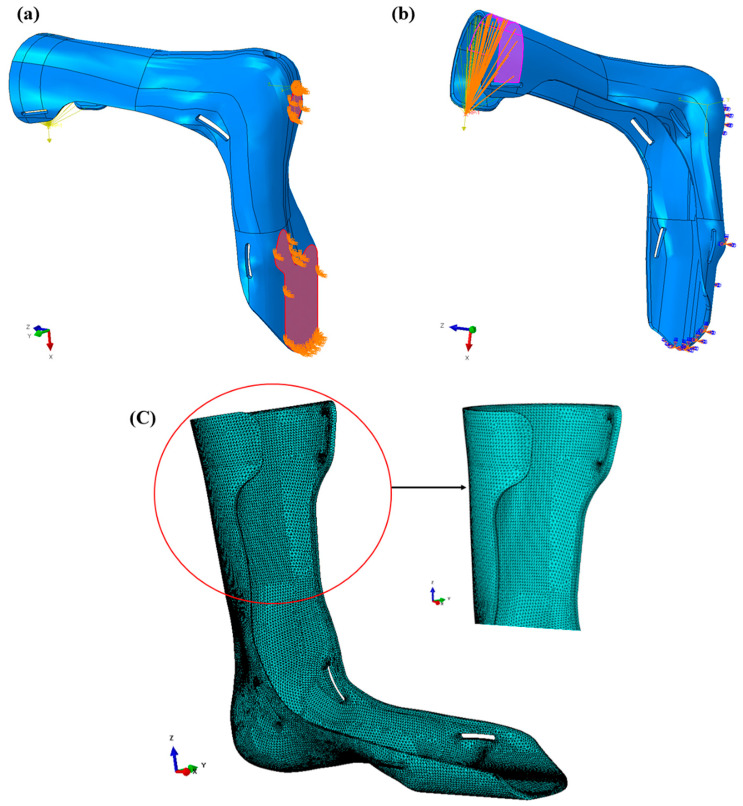
FEA model with (**a**) generated fixed supports, (**b**) loading condition for better replication of experimental testing, and (**c**) mesh geometry.

A computer with a 3.07 GHz Intel Core i7-9300HF processor and 24 GB of RAM was used to evaluate the FEA models of AFOs. The properties of materials used in the simulation of AFOs were set using the ASTM standard sample results, and those used for the second simulation were calculated using the outcome values of Equation (2). The simulation replicated the strength testing of two types of AFOs used in this study. The stationary boundary conditions were placed at the ground contact section of the foot part to replicate the real condition that a patient could feel while walking. The load was applied to the uppermost part of the AFOs for the replication of force exerted on the calf strap by the patient while walking. This was carried out to mimic where the AFO samples were held during the strength testing and to duplicate the areas where the patient’s strapping restricts the AFO’s range of motion. The AFO’s STL file was imported into the SIMULIA Abaqus version 6.12-2 FEA software to create the volumetric model and mesh and apply boundary conditions. Figure 4 shows the 3D FEA model with supports and loading condition. The mesh required to run the simulation was modeled assuming that the manufactured samples were homogeneous and anisotropic at any cross-section. In order to best fit this shape, the tet (tetrahedral) C3D10 element with a 1.4 mm size was used. To search for 3D stress, an element type with a quadratic geometric order as well as improvisational settings of surface-stress formulations were adopted. As part of the meshing process, 271,945 elements were seeded onto the 276 faces of the geometry with 435,545 nodes prior to simulation. The boundary conditions were imposed so that they could simulate the static loads that were applied during the experimental tests. The simulations were designed to replicate the real experimental strength testing of the AFO samples. Although a perfect match was impossible due to testing equipment limitations, the modeling objective was to simulate the stress and strains an individual would experience while wearing the AFO. The force was concentrated on the loading device shown in Figure 5a,b. The mesh geometry considered for the calculation can be seen in Figure 5c. In this analysis, there were 15 warnings, which is 0.00551582%, and therefore, there was no analysis error. In order to guarantee mesh independency, the analysis element size was continuously reduced to 1.2 mm (number of elements 312,579) to achieve constant displacement results. Mesh independency graph can be found in supplementary materials, Figure S1. In this case, a constant trend was observed after the 1.4 mm (number of elements 271,945) element size. The AFO was constrained by the boundary conditions in the initial step, but it did not experience any load, as seen by its undamaged shape. The load location was approximately the same where the force was applied in the strength testing experiments. This was accomplished using a constraint that allowed the loading surface to be connected to a point in space where a directional force was applied. Only x-direction displacements were produced by simulating the device. The elastic modulus and Poisson’s values of the materials, which were obtained from tensile tests of 3D-printed ASTM/ISO D638 samples, were used to simulate the AFOs of both materials in this section. The calculated values shown in Table 2 were very similar to those seen in previous studies [37].

In this case, dual simulation means that the simulation was run twice with different modulus values. The first simulation with standard modulus values and the outcomes along with experimental displacements were used in Equation (2) to calculate the real elastic modulus for 3D-printed AFOs. Second, seeding these real calculated modulus values in the material properties section, AFOs were simulated again to achieve realistic results. Knowing that the mass and geometric values were the same in the simulation and experiment and that the force employed in the simulation was equal to the force obtained during the experiment, the AFOs’ loading force and displacement values were computed and compared to the outcomes of the experimental strength testing.

## 3. Results

### 3.1. Manufacturing Results

The manufacturing of orthoses with PLA did not pose any difficulties, but 3D printing with PLA-C filaments showed difficulties and errors in manufacturing AFOs. However, most of the challenges and errors were caused by the operator and were corrected as soon as they were discovered. For instance, a bad calibration of the bed and improper cleaning of the bed and nozzle as well as clogged nozzles were some of the errors. A widening of the nozzle diameter was observed during the printing of PLA-C based AFOs. Still, it did not impact the manufacturing process, as no defects were noticed in the final product. There were several potential causes for the widening of nozzles, one being the presence of CF in the filament, which acts as a rubbing agent when processed. This phenomenon opens a new field of research for scientists and researchers working within the 3D printing field. The weight of the final 3D-printed AFOs was measured (Table 3), and it was found that PLA-C produces a lighter AFO than PLA.

### 3.2. Mechanical Strength Test and FEA Comparison

**Figure 6:**
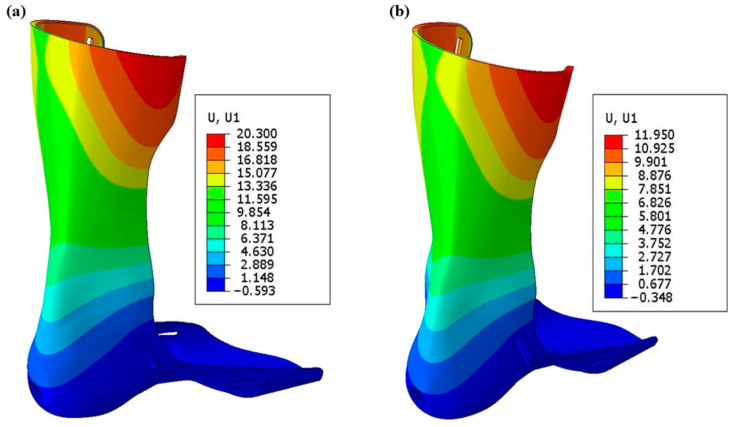
Simulated deformation distribution of (**a**) PLA and (**b**) PLA-C.

In order to verify the accuracy of the FEA results, experimental mechanical testing was performed to ascertain the force versus displacement relationships of each AFO. A comprehensive comparison between the F-d relationship generated by strength testing and from the FEA of all AFOs was conducted to validate the FE model. Figure 6 shows that the predicted results of the AFOs match well with the experimental results. The FEA predicted that the displacements in the applied load of 160 N for PLA-based AFO and PLA-C-based AFOs at the loading point were very near to the experimental values (refer to Table 4).

### 3.3. Energy Analysis

**Figure 7:**
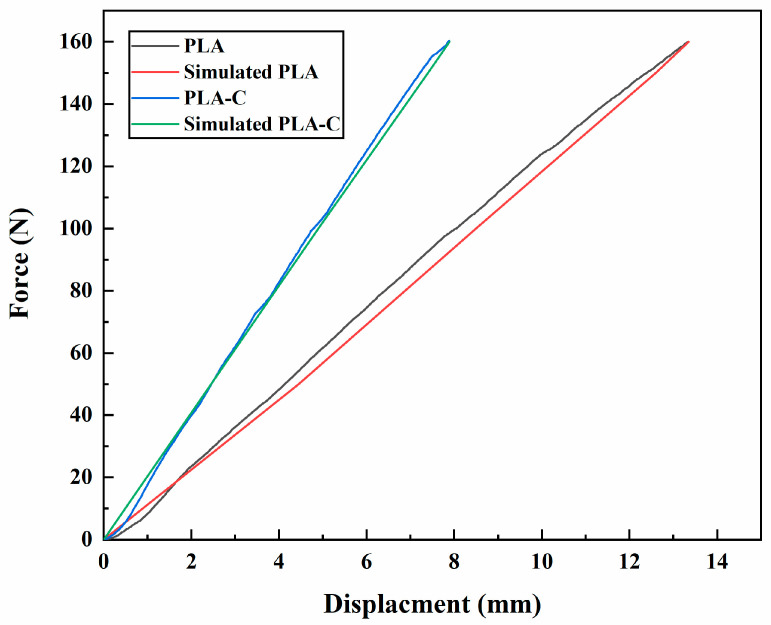
Force–displacement curves for both AFOs as determined by FEA and experimental testing.

**Figure 8:**
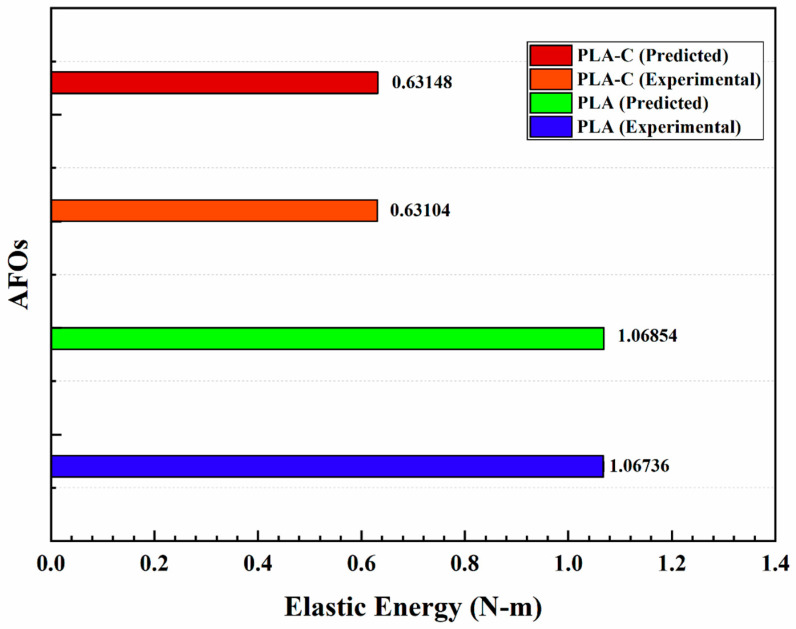
Predicted and experimental energy of both types of AFOs.

The FEA model’s F-d curves and the results of the experimental tests agreed well. Therefore, by calculating the area under the loading curves, it was possible to precisely determine the elastic energy of the orthoses. Figure 7 displays the loading curves for both AFOs as determined by FEA and experimental testing (detailed values of displacements at different forces can be found in supplementary material, Tables S1 and S2, complemented with Figures S2 and S3). Both types of AFOs showed a relatively linear F-d loading curve. The PLA-C showed a high stiffness in comparison to the PLA-based AFO. Figure 8 shows the predicted and experimental energies of both types of AFOs, where it can be seen that the FEA models made accurate predictions of energy. The FEA-predicted energy values for PLA- and PLA-C-based AFOs showed minute errors of 0.11% and 0.07%, respectively, with the experimental results.

### 3.4. Fracture Analysis

**Figure 9:**
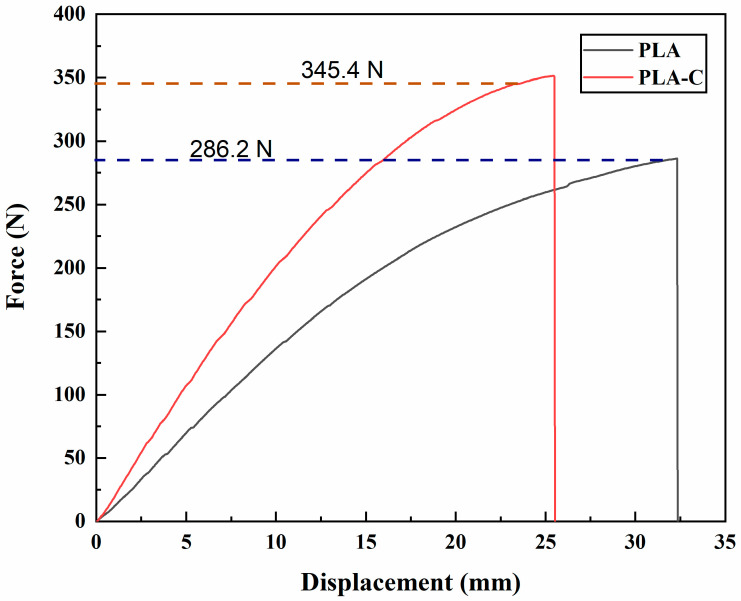
Force–displacement curves for both PLA- and PLA-C-based AFOs until fracture.

**Figure 10:**
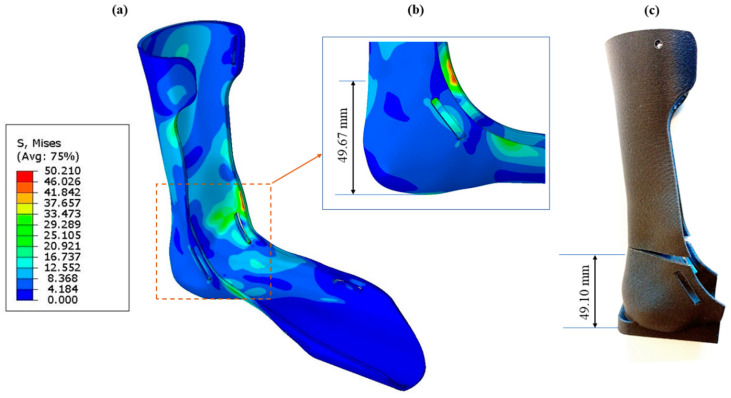
For PLA-C-based AFO, (**a**) simulated stress distribution, (**b**) high-stress location from sole in simulated result, and (**c**) location of fracture during experimentation.

AFOs were loaded until both failed structurally after completing the mechanical testing. In this study, this fracture analysis was conducted to determine the maximum load that the AFOs could withstand and to validate the FEA model. The fracture occurred at a load of 286.2 N for PLA and 345.4 N for PLA-C, and the fracture for PLA-C can be seen in Figure 9. During the FEA model simulation, the load was increased to 286.2 and 345.4 N for PLA and PLA-C, respectively, based on the mechanical test results. The FEA results of the lower shank’s Mises stress distribution are shown in Figure 10a for the PLA-C-based AFO. The maximum stress in the lower shank area for PLA- and PLA-C-based AFOs is tabulated below (Table 5). For PLA-C, the predicted fracture distance from the sole was 49.67 mm, which was extremely close to the experimental result of 49.10 mm (Figure 10b,c). Table 5 shows that the experimental and predicted fracture locations, with respect to the sole for both types of AFOs, had minute error percentages.

## 4. Discussion

An AFO made with 3D printing is lighter than an AFO made with conventional materials. The cost of production of these is significantly lower than that of conventionally produced products. No cracks were observed during the testing of the AFOs for the load of 160 N after five repetitions. This is the approximately the amount of force a 16 kg child exerts when walking. Both AFOs displayed linear behavior with this load, which means they were still under their elastic limits. Within this limit, the materials showed completely elastic behavior, meaning the deflection returned almost to its original state once the load was removed. Thus, it can be stated that AFOs are durable for the child. Due to the fact that static loads were taken into account in this study, it cannot be claimed that the AFO would be durable while jumping, running, or playing. A possible future extension of this study would be to examine dynamic loading conditions. The calculation of elastic potential energy was taken into account in this study to generate a better understanding of the phenomenon. The PLA-C-based AFOs showed less elastic energy, which means there was less deflection while applying 160 N. Due to the fact that there was less stored energy, the PLA-C-based AFOs were more rigid than the PLA-based ones, which is why they should be considered over PLA-based ones. The experiment showed that CF enhanced the strength and durability of PLA when mixed with it. Researchers have observed the same results while experimenting with CF-reinforced thermoplastics for the production of AFOs. It helped to enhance the accuracy of the FEA model for these orthoses by considering the fixation of the lower part and the loading at the other end. The minute differences observed between the experimental and predicted elastic energy values show that the model planning was accurate. As a result of these accurate predictions for both AFOs, this model is capable of assisting in the optimization of AFO fabrication techniques. The three-step model helped in making predictions more precise because, at each step, a high degree of precision was observed. Each step represented a different range of loads in this study. This comparison was made simpler by using Equation (2), which is well-suited to predicting the actual elastic modulus of the fabricated structures. The matching of both results is sufficient in and of itself to demonstrate the acceptability and accuracy of the equation derived from the proportionality of the loading curve. This study also used fracture analysis to determine the stress value at the fracture point by comparing the experimental and simulation results. Due to the complex shape of the AFO, it was difficult to produce a stress–strain plot during the experiments. However, yield stress values are of technical significance when choosing materials and designing for applications since they can simply be used to determine the maximum load that a given material can withstand. Therefore, a smart method for developing and choosing materials in accordance with the advised load values could be offered by this fracture analysis of AFOs. Although the stress levels at the fracture point recovered from the simulation may not exactly match the stress created during testing, it can be claimed that they represent the genuine value because the fracture occurred in the same place in both. The highest yield stress point agreement between the simulation and experiment demonstrates the model’s genuine capacity for outcome prediction. The model and Equation (2) employed in the study may be the most effective tools for choosing materials in accordance with specifications without squandering resources or testing time.

However, this study is not easily comparable with previous works because of different study parameters, such as different loading conditions and design. Rather than this, Table 6 could provide a concise summary of recent studies to make it easier for readers to make a significant interpretation of the variable design and loads for respective displacements and stresses.

## 5. Conclusions

The study concluded, on the basis of the elastic energy calculation extracted from F-d curves, that the 3D-printed PLA-C-based AFOs were stiffer than the PLA-based ones. The proportionality equation enabled the prediction of the real values of printed AFOs by using the experimental deflection and modulus values of ASTM standard samples. The design modification and fixture design for experimental strength testing were performed to resemble an exact replication of the FEA model. The matching of outcomes from experiments and simulation confirmed that the model is well-established and can be used for other materials. Fracture stress identification makes the results strong for the proper selection of materials for AFOs based on the advised load. For PLA-C, the predicted fracture distance from the sole was 49.67 mm, which was close to the experimental result of 49.10 mm. Similarly, the predicted distance for PLA also matched well with the experimental value. The authors claim that the model and proposed experimental setup will be applicable for almost every material. Future studies will focus on use of other materials, including popular filaments such as ABS, PETG, and PA12 as well as more advanced materials such as PEEK and PC.

Finally, it has been concluded that such highly accurate models will lead to improved AFO fabrication without being wasteful of resources and time. In the future, this study may extend to explore AFOs made from other 3D-printed CF-reinforced thermoplastics that are compatible with FDM under static and dynamic loading conditions. A dynamic loading condition simulation model with loading angle variation opens up a whole new horizon for future study.

## Figures and Tables

**Table 1:** The 3D printing parameters and weights of printed samples.

Material	Extrusion Temperature (°C)	Infill (%)	Layer Thickness (mm)
PLA	210	100	0.29
PLA-C	215	100	0.29

**Table 2:** Material properties obtained from tensile testing of ASTM samples.

Material	Young’s Modulus (MPa)	Poisson’s Ratio
PLA	3374	0.33
PLA-C	3926	0.40

**Table 3:** Weight of 3D-printed AFOs.

Material	AFO’s Weight (in Grams)
PLA	84.906 ± 0.071
PLA-C	77.038 ± 0.052

**Table 4:** Experimental and predicted displacements of AFOs at 160 N load.

Material	Experimental Displacement (mm)	Predicted Displacement (mm)	Error (%)
PLA	7.888	7.893	0.069
PLA-C	13.342	13.356	0.105

**Table 5:** Maximum stress generated at lower shank of AFOs and distance of fracture location from sole.

Material	Maximum Stress Developed (MPa)	Experimental Fracture Location (mm)	Predicted Fracture Location (mm)	Error (%)
PLA	39.058	49.60	50.11	1.017
PLA-C	50.210	49.10	49.67	1.147

**Table 6:** Comparative study between the present work and previously published articles on AFOs.

Materials	Process	Load (N)	Displacement (mm)	Maximum Stress Developed (MPa)
This work (PLA-C)	3D-Printed	345	7.89	50.21
Acrylonitrile butadiene styrene [38]	3D-Printed	657	0.27	9.50
PLA and Nylon-12 [39]	3D-Printed	490	1.42	81.35
Polypropylene [38]	3D-Printed	657	0.37	10.00
PLA [23]	3D-Printed	300	2.66	8.32
PLA [40]	3D-Printed	50	-	25.00
Thermoplastic with carbon fiber [41]	Casting	328	2.10	-
Polypropylene [42]	Casting	750	-	12.00

## Data Availability

Not applicable.

## Supplementary Materials

The following supporting information can be downloaded at: https://www.mdpi.com/article/10.3390/ma15176130/s1, Figure S1: Mesh independency graph between displacement versus number of elements; Figure S2: Predicted displacement values at (a) 50 N and (b) 150 N for PLA-based AFOs; Figure S3: Predicted displacement values at (a) 50 N and (b) 150 N for PLA-C-based AFOs; Table S1: Simulation and experimental displacements for AFOs manufactured using PLA at different forces; Table S2: Simulation and experimental displacements for AFOs manufactured using PLA-C at different forces.
