# Supplementary material for: Numerical and Experimental Mechanical Analysis of Additively Manufactured Ankle–Foot Orthoses
Source: Materials (Basel). 2022 Sep 3;15(17):6130. doi: 10.3390/ma15176130 (PMC9457881; doi:10.3390/ma15176130)
Supplement: Supplementary file 1 [file materials-15-06130-s001.zip › materials-1853653-supplementary.pdf]

Supplementary

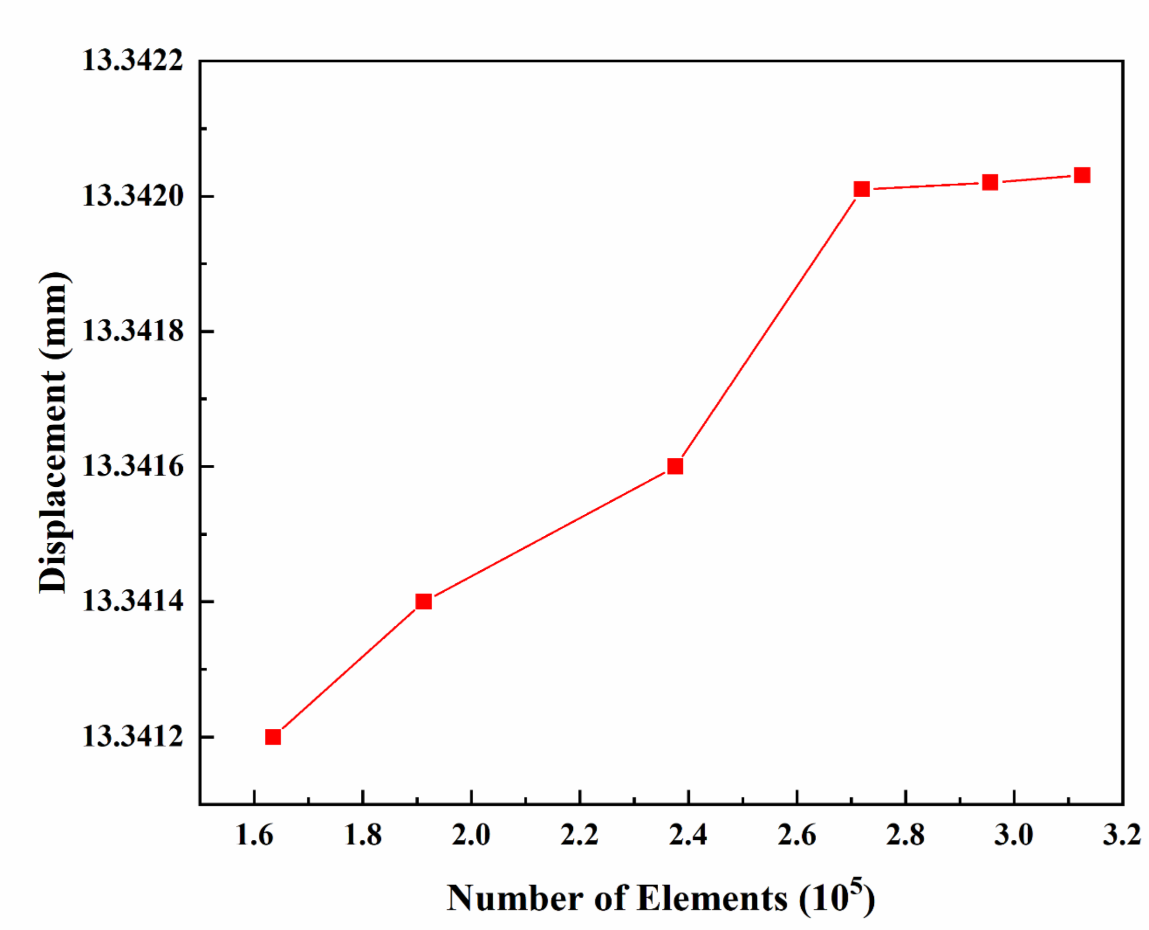

Figure S1: Mesh independency graph between displacement versus number of elements.

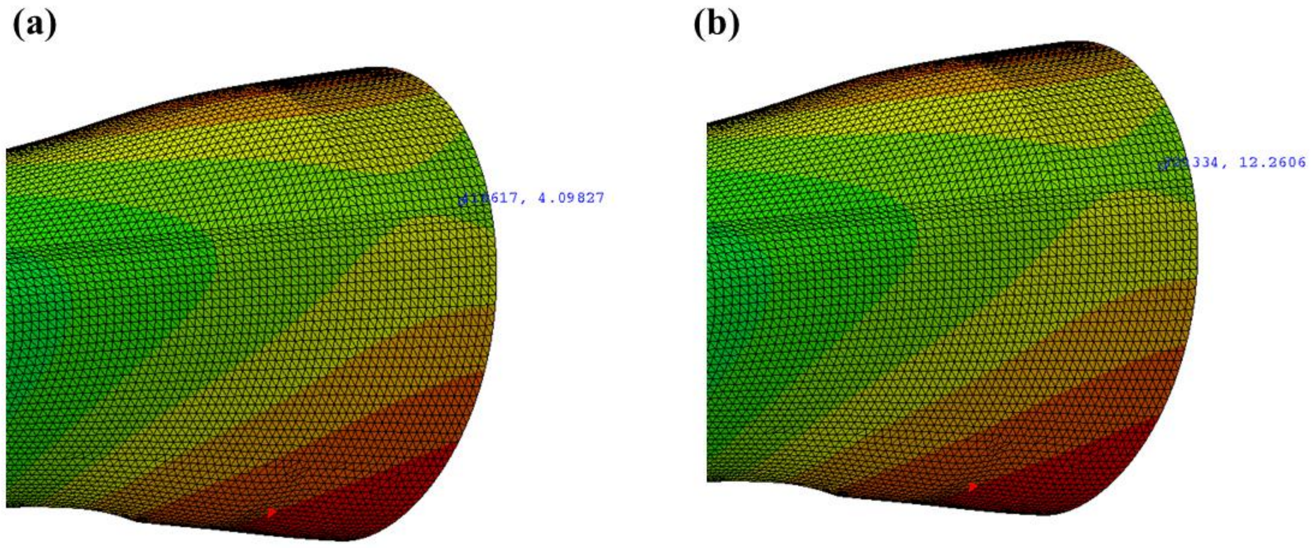

Figure S2: Predicted displacement values at (a) 50 N and (b) 150 N for PLA-based AFOs.

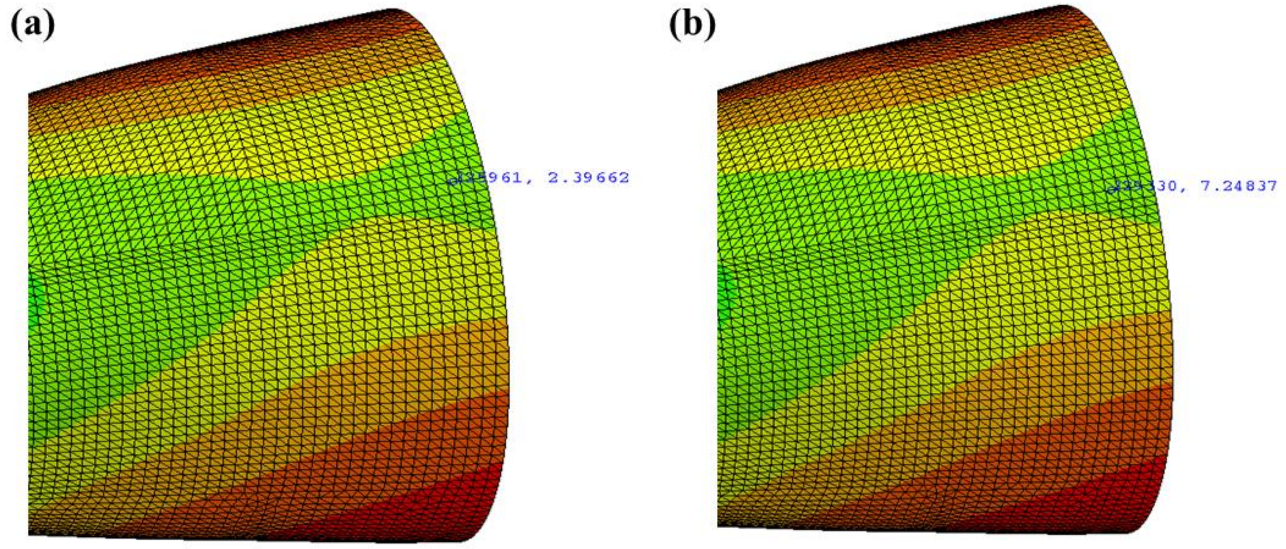

Figure S3: Predicted displacement values at (a) 50 N and (b) 150 N for PLA-C-based AFOs.

Table S1: Simulation and experimental displacements for AFOs manufactured using PLA at different forces.

Force (N)	Displacement (Experimental) (mm)	Displacement (Predicted) (mm)	Error (%)
50	4.012	4.098	2.098
100	8.047	8.513	5.473
150	12.010	12.260	2.039
160	13.342	13.356	0.105

Table S2: Simulation and experimental displacements for AFOs manufactured using PLA-C at different forces.

Force (N)	Displacement (Experimental) (mm)	Displacement (Predicted) (mm)	Error (%)
50	2.462	2.396	2.754
100	4.792	4.912	2.443
150	7.221	7.248	0.373
160	7.888	7.893	0.069
